# Predicting Cartographic Symbol Location with Eye-Tracking Data and Machine Learning Approach

**DOI:** 10.3390/jemr18040035

**Published:** 2025-08-07

**Authors:** Paweł Cybulski

**Affiliations:** Department of Cartography and Geomatics, Adam Mickiewicz University Poznan, 61-712 Poznan, Poland; p.cybulski@amu.edu.pl; Tel.: +48-61-829-6251

**Keywords:** machine learning, cartography, eye tracking, visual search, location prediction, map reading

## Abstract

Visual search is a core component of map reading, influenced by both cartographic design and human perceptual processes. This study investigates whether the location of a target cartographic symbol—central or peripheral—can be predicted using eye-tracking data and machine learning techniques. Two datasets were analyzed, each derived from separate studies involving visual search tasks with varying map characteristics. A comprehensive set of eye movement features, including fixation duration, saccade amplitude, and gaze dispersion, were extracted and standardized. Feature selection and polynomial interaction terms were applied to enhance model performance. Twelve supervised classification algorithms were tested, including Random Forest, Gradient Boosting, and Support Vector Machines. The models were evaluated using accuracy, precision, recall, F1-score, and ROC-AUC. Results show that models trained on the first dataset achieved higher accuracy and class separation, with AdaBoost and Gradient Boosting performing best (accuracy = 0.822; ROC-AUC > 0.86). In contrast, the second dataset presented greater classification challenges, despite high recall in some models. Feature importance analysis revealed that fixation standard deviation as a proxy for gaze dispersion, particularly along the vertical axis, was the most predictive metric. These findings suggest that gaze behavior can reliably indicate the spatial focus of visual search, providing valuable insight for the development of adaptive, gaze-aware cartographic interfaces.

## 1. Introduction

The visual search process on a map is one of the fundamental activities of map reading, enabling users to efficiently locate, identify, and interpret relevant spatial information [1]. This cognitive task involves scanning the map for specific symbols, patterns, or geographic features, and is often influenced by factors such as symbol design, contrast, color, and spatial arrangement [2]. The effectiveness of visual search on maps is shaped by both human perceptual abilities and cartographic design, making it a key factor in user experience. Recognizing this, research in cartography and GIScience has increasingly focused on adapting maps to the user’s context [3,4]. Future map applications aim to go beyond static representations, offering dynamic visualizations that adapt to human activity and even cognitive states. By tailoring the presentation of information to users’ needs in real time, these advancements seek to improve search efficiency, engagement, and overall usability in map-based tasks [5,6]. For example, by analyzing users’ eye movement parameters, future map applications could predict or recognize the location of target cartographic symbols, further enhancing adaptive visualization strategies.

In visual search tasks during map reading, users often look for a target symbol representing a feature or place of interest, such as a shop or restaurant [7,8]. The challenge of recognizing the target symbol’s location based on eye movement parameters stems from the limited understanding of the cognitive processes involved in map reading—particularly during visual search. This is further complicated by the fact that different users may apply distinct visual strategies and reading patterns [2,9].

In recent years, the use of eye-tracking (ET) technology to recognize and analyze visual activities has gained significant attention, particularly in studies involving map reading and visual search [10,11]. Eye tracking allows for the precise monitoring of where and for how long a user focuses their gaze, providing valuable insights into attention allocation and cognitive processing during map interaction [12,13]. By analyzing eye movement patterns—such as fixations, saccades, and gaze duration—researchers can infer users’ levels of attention, cognitive load, and engagement with specific map elements, including symbols, geographic features, or spatial relationships [14,15]. Gaze dispersion—the spatial distribution of visual fixations—is increasingly used as a proxy for cognitive load, attention allocation, and decision-making processes [16,17]. Research consistently demonstrates that higher cognitive load, complex decision-making, or divided attention are associated with changes in gaze dispersion patterns [18]. In particular, studies in cartography have leveraged gaze dispersion metrics to investigate how users process spatial information, interpret map features, and navigate complex visual displays, highlighting the relevance of both vertical and horizontal dispersion in tasks requiring spatial cognition and visual exploration [19,20].

The goal of this study is to leverage machine learning techniques to predict the location of target symbols on a map—specifically, whether they are situated in peripheral or central areas—based on selected eye-tracking metrics. By analyzing eye movement data such as fixation duration, saccade length, pupil size, and fixation dispersion, the study aims to develop a model that can classify the position of a target symbol in relation to the user’s gaze behavior. Machine learning algorithms will be trained on eye-tracking data to identify patterns and predict whether the symbol is located in the central or peripheral region of the map, offering new insights into the relationship between gaze behavior and spatial perception during map reading.

In addition, the study seeks to compare two distinct experimental datasets to assess which is more suitable for predictive modeling, considering differences in task design, class balance, and data complexity.

## 2. Related Work

In the study by Kiefer et al. [21], eye-tracking data was used to recognize user activities on cartographic maps. The researchers collected gaze recordings and spatiotemporal eye movement features from 17 participants performing six different tasks. Using a Support Vector Machine (SVM) classifier, they achieved a classification accuracy of 78%, successfully distinguishing activities such as free exploration, global search, and route planning. These results demonstrate that eye-tracking metrics can effectively predict user activity, contributing to the development of gaze-assisted map interfaces.

Liao et al. [22] explored the use of machine learning to infer user tasks in real-world pedestrian navigation scenarios based on eye-tracking data. The study involved 38 participants who performed five predefined navigation tasks, including self-localization, environment target search, map target search, route memorization, and walking to a destination. Eye movement data were collected using SMI eye-tracking glasses, and features such as fixation density, saccade direction, and saccade encoding were extracted. A Random Forest classifier was trained to classify the tasks, achieving an overall accuracy of 67%. The study found that statistical eye movement features and saccade encoding features were particularly useful for distinguishing among tasks. These findings suggest that eye-tracking data can be leveraged to predict user tasks in navigation, with implications for the development of adaptive navigation systems that provide task-specific assistance.

In the study by Qin et al. [23], the authors investigated the recognition of map activities using a combination of eye-tracking and electroencephalography (EEG) data. The study focused on four common map tasks: global search, distance comparison, route following, and route planning. Eye-tracking data, including fixation and saccade metrics, were used to capture visual attention patterns, while EEG data provided insights into cognitive processes. The authors evaluated 11 different classifiers, with LightGBM achieving the highest accuracy of 87.9% when combining eye tracking and EEG features. Saccade features (e.g., velocity, amplitude, and duration) were found to be the most important for distinguishing map activities, as they reflect users’ visual search strategies. The results suggest that eye-tracking metrics—particularly those related to saccades—are highly effective in predicting user tasks on maps, with potential applications in adaptive map design and real-time navigation systems.

Keskin and Kettunen [24] conducted a review exploring the potential of combining eye-tracking and machine learning for interactive geovisual exploration, with a particular focus on gaze-aware interactive map systems (GAIMSs). The study examined the use of eye tracking to observe user behavior and enable gaze-based interaction with geovisualizations, emphasizing the adaptation of map designs based on users’ attentional feedback. The authors proposed a framework in which eye-tracking data highlights vector map features that receive the most attention, while machine learning is used to detect similar features across the map. This approach aims to enhance spatial understanding and assist users in exploring unfamiliar or complex geospatial data. The study highlights the need for further research on gaze-based interaction and machine learning for processing vector geospatial data to improve the usability of geovisualizations.

While previous research has demonstrated that eye-tracking data can be used to recognize general user activities on maps, little attention has been given to how specific aspects of gaze behavior, such as gaze dispersion along vertical and horizontal axes, relate to the prediction of target locations. While users can locate map symbols directly, predicting whether a symbol lies in the central or peripheral region based solely on eye movement data provides valuable insight into visual search strategies and attentional demands. This goes beyond simple classification—it reveals how users interact with different spatial areas. Peripheral symbols, for example, often elicit broader scan paths and greater gaze dispersion, indicating higher cognitive load. Such predictions can serve as proxies for task complexity and inform adaptive map interfaces. Gaze-aware systems could use this information to adjust content in real time, e.g., by magnifying peripheral zones or emphasizing target areas.

However, most cartographic studies focus on overall user activity classification or general attention patterns rather than predicting spatial attributes of the target itself. This study addresses this gap by investigating whether gaze behavior—particularly the extent of vertical and horizontal gaze dispersion during visual search—can predict whether a searched target symbol is located in peripheral or central areas of a map.

## 3. Materials and Methods

**Figure 1:**
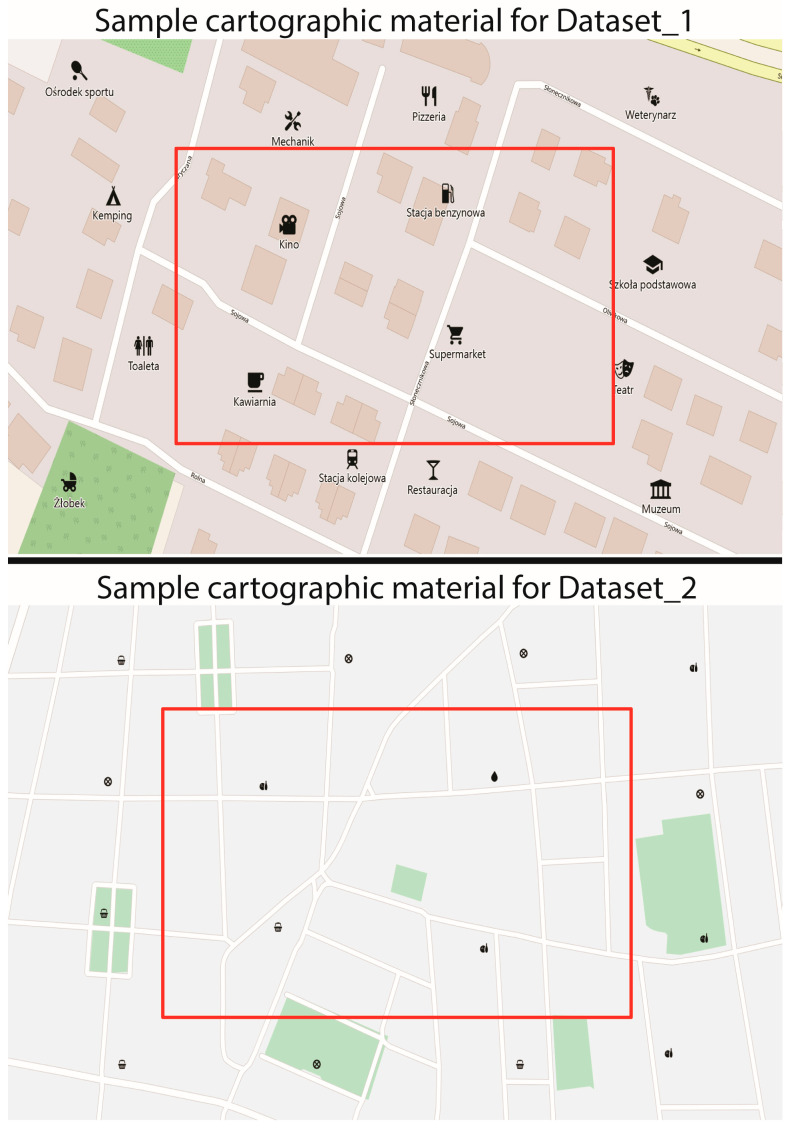
Sample cartographic materials used in both studies. The red rectangle represents the central area of visual search, while the remaining area is considered peripheral. Source: https://doi.org/10.7910/DVN/IUK4SZ.

The data for this study comes from two previous experiments. The first study [25], referred to in the text as Dataset_1, focused on visual search for a target cartographic symbol labeled in either Chinese or Polish, where the target could appear in the central or peripheral area of the map. The second study [26], referred to as Dataset_2, involved searching for a target symbol among unlabeled distractor symbols with varying levels of graphical similarity. Both studies used the same number of distractor symbols and only one target symbol, with a similar number of participants recruited from the same institution. However, the studies differed in their design regarding the definition of central and peripheral areas, symbol size, and the eye-tracking devices used. Although both tasks involved searching for a target symbol without time constraints, the cognitive demands may vary slightly (Figure 1).

In Dataset 1, each participant viewed 24 maps, of which 8 contained central targets, 8 peripheral targets, and 8 maps had no target at all. For this study, only maps containing a target symbol were used, yielding a balanced dataset: 50% central, 50% peripheral. A model always predicting the majority class in this dataset would therefore achieve a baseline accuracy of 50%. In Dataset 2, the target symbol was always present, with its position randomly assigned across five predefined regions (central + four peripheral quadrants). Out of the 60 stimuli, 12 placed the target centrally and 48 peripherally, resulting in a natural class imbalance: 20% central, 80% peripheral.

Dataset 1 involved 38 geography students (21 males, 17 females; aged 22–24, mean age 23 ± 0.8) from Adam Mickiewicz University, Poznań. Eye movements were recorded using a Tobii X2-60 eye tracker (60 Hz sampling rate, 0.5° spatial accuracy) with a 1980 × 1080 px laptop display. Participants sat ~60 cm from the screen, and calibration was performed using a 9-point procedure (average gaze sample score: 92% ± 1.5%). Each participant viewed 24 maps, resulting in a total of 918 individual eye movement recordings. Dataset 2 included 33 participants (16 males, 16 females, 1 other; aged 19–23, mean age 21.5 ± 3.2), also from Adam Mickiewicz University, with backgrounds in geography and cartography. Eye tracking was conducted with an EyeLink 1000 Plus (SR Research Ltd., Ottawa, ON, Canada) (2000 Hz sampling rate), using chin and forehead rests for head stabilization. The stimulus was displayed on a 21-inch monitor (1920 × 1080 px) viewed at a ~90 cm distance, with the eye tracker positioned ~55 cm from the participant. Each participant analyzed 60 maps, generating a total of 1980 eye movement recordings.

Therefore, separate machine learning models were developed for each dataset. This approach allows the models to be tailored to the unique characteristics of each study, potentially improving performance and result interpretability.

### 3.1. Eye-Tracking Features

To capture the characteristics of eye movements, an identical set of features was created for both datasets, based on the studies by Kiefer et al. [22] and Qin et al. [23], with slight modifications. In total, 32 features and statistical measures were included in the analysis.

(a) Fixation-based features

Since fixations indicate the user’s visual attention [27,28], fixation duration was analyzed along with basic statistical measures, including mean, median, maximum, minimum, standard deviation, skewness, and kurtosis. Additionally, fixation count and the standard deviations of fixation positions in the horizontal (X) and vertical (Y) directions were included as proxies for gaze dispersion, a common measure for spatial spread of visual attention during search tasks.

(b) Saccadic-based features

Saccades represent the movement of visual attention across different areas of the map, indicating that users are actively engaged in the visual search process [29,30]. Three saccadic features were used: saccade count, saccade amplitude, and saccade velocity. For the latter two, basic statistical measures were calculated, including mean, median, maximum, minimum, standard deviation, skewness, and kurtosis.

(c) Pupil-based features

Pupil diameter is often associated with the cognitive load experienced by users [31,32], but it may also reflect emotional processing [33]. Therefore, pupil size was analyzed, and basic statistics were calculated for both fixation and saccade features.

### 3.2. Data Processing

Data standardization, also known as feature scaling, is an essential step in the machine learning process when predicting cartographic symbol locations using eye-tracking data [34]. The procedure standardizes the data by transforming all numerical features to have a zero mean (*μ* = 0) and unit variance (*σ*^2^ = 1). This ensures that each feature contributes equally to the model, which is particularly important for distance-based models like K-Nearest Neighbors (KNN), Support Vector Machines (SVM), and Neural Networks. Without standardization, features with larger magnitudes could dominate the model, leading to biased predictions. For example, if the mean fixation duration is 500 ms and the mean saccadic amplitude is 4 degrees, without scaling, the model might give more importance to fixation duration simply due to its higher numerical value. By applying standardization, both features are brought to the same scale, allowing the model to treat them equally, thus improving its accuracy and performance.

Feature selection plays a critical role in enhancing model performance and computational efficiency by identifying the most relevant features for prediction tasks [35]. This study employs a feature selection method that evaluates the statistical significance of each feature using the Analysis of Variance (ANOVA) F-statistic. This approach helps identify the top 10 features most strongly associated with the target variable. The F-statistic measures the variance between each feature and the target, with higher values indicating greater relevance. By selecting only the most significant features, we reduce the dimensionality of the dataset, which enhances model training efficiency and may improve prediction accuracy. To assess the effectiveness of this feature selection method, we compare it with alternative techniques, such as variance-based selection and mutual information-based selection, and analyze their impact on model performance.

In this study, the dataset was split into two parts: a training set (80% randomly selected) and a test set (20% of the data not used in the training) to ensure that the model can be trained and evaluated effectively. By training the model on one set of data (the training set) and evaluating it on a different set (the test set), we can ensure that the model does not overfit to the training data. To prevent data leakage, the training and test sets were constructed at the participant level. That is, all trials from a given participant were assigned exclusively to either the training or the test set. This approach avoids overlap in gaze behavior patterns across sets and ensures that model evaluation reflects generalizability to unseen participants.

### 3.3. Machine Learning Model Selection

Based on previous studies [23], this research incorporated 12 classifiers, which are types of models used to assign labels to data points based on their features (Logistic Regression, K-Nearest Neighbors, Linear Discriminant Analysis, Support Vector, Decision Tree, Random Forest, AdaBoost, Gradient Boosting, Multi-Layer Perceptron, XGBoost, Voting Ensemble, and LightGBM). Specifically, these classifiers are used for supervised tasks, where the goal is to predict a categorical label (in the case of this study, the label is “Location,” which could be either central or peripheral). All models include several techniques to improve classification performance.

To account for potential non-linear relationships between variables, polynomial feature transformation was applied in this study. This process generates new interaction terms that capture relationships between pairs of original features, allowing the models to identify complex dependencies that may not be evident through individual features alone [36]. However, to maintain interpretability and avoid excessive complexity, these interaction terms were not retained as composite features in the final analysis. Instead, they were decomposed back into their individual components, ensuring that only the original features were used in the evaluation of feature importance and selection [37]. This approach strikes a balance between improving model performance through richer training data and preserving the clarity of feature interpretation. For each dataset, the top six features ranked by F-statistic were selected and used for model training. These features varied slightly across models due to interactions introduced by polynomial terms and internal selection processes. A full list of features retained for each model is provided in Table 6 and Table 7 further in the text (Section 4) for Dataset_1 and Dataset_2, respectively.

To enhance the performance and generalization ability of the model, hyperparameter tuning was applied. Optimizing these parameters is crucial to achieving the best balance between underfitting (where the model is too simple to capture patterns) and overfitting (where the model is too complex and memorizes the training data) [37]. In this study, hyperparameter tuning was performed to determine the optimal configuration for the Random Forest model. Key parameters, such as the number of trees in the forest [100, 200], the maximum depth of each tree [None, 10, 20], and the minimum number of samples required for splitting nodes [2, 5] or forming leaf nodes [1, 2], were systematically tested. By evaluating multiple configurations and selecting the best-performing combination, this approach ensures that the final model is both accurate and generalizable to unseen data, leading to improved prediction performance [38].

Since a majority-class classifier would achieve an 80% accuracy baseline in Dataset 2, SMOTE (Synthetic Minority Over-sampling Technique) was applied to the training data [39], producing a perfectly balanced dataset (50/50) for training purposes. The test data retained the original class distribution to ensure fair performance evaluation.

## 4. Results

To comprehensively evaluate the model’s performance, multiple metrics were used, including accuracy, precision, recall, F1-score, and ROC-AUC (Receiver Operating Characteristic Area Under the Curve) [40].

The two datasets used in this study differ in their design, balance, and visual search complexity. Dataset_1 features a balanced design with a 50/50 central-to-peripheral distribution, whereas Dataset_2 reflects a naturalistic, imbalanced setting (20/80 central-to-peripheral). Moreover, the symbol similarity, language familiarity, and map layout differ across the datasets. For this reason, results are presented comparatively to assess which dataset structure is more suitable for predictive modeling using eye-tracking metrics. This side-by-side comparison helps identify not only model performance differences but also the extent to which dataset complexity affects gaze-based prediction in cartographic tasks.

### 4.1. Accuracy

Accuracy is defined as the ratio of correct predictions to the total number of predictions and provides a general measure of the model’s overall correctness.

For Dataset 1, the highest accuracy was achieved by AdaBoost and Gradient Boosting (both at 0.822), indicating strong predictive performance. Other models, with accuracies ranging from 0.813 to 0.804, demonstrated reasonable but not outstanding performance. The LDA model, with the lowest accuracy (0.719), falls into an average performance range, highlighting potential areas for improvement.

For Dataset 2, the best accuracy was obtained by LGBM (0.773), followed closely by the Voting Ensemble and Random Forest models (both at 0.770), indicating moderate classification performance. The lowest-performing model, again LDA, achieved an accuracy of 0.652, suggesting limited predictive reliability.

Overall, the results reflect moderate model performance across both datasets, with AdaBoost and Gradient Boosting in Dataset 1 emerging as the most promising. However, accuracies below 0.70 indicate that further enhancements—such as refined feature engineering or more advanced hyperparameter optimization—may be required to improve classification robustness. This suggests that Dataset 1 contains more informative features and clearer patterns, making classification between peripheral and central locations easier. A summary of model accuracies is presented in Table 1.

### 4.2. Precision

Precision (also known as the Positive Predictive Value) measures how many of the cases the model predicted as peripheral are actually correct. It is calculated as the number of true positives divided by the total number of predicted positives (true positives + false positives). High precision means the model makes few false alarms.

The precision results for both datasets reveal strong model performance in identifying peripheral and central eye-tracking instances while searching for cartographic symbols, with several models demonstrating high reliability in minimizing false positives. For Dataset_1, the AdaBoost model achieved the highest precision (0.90), indicating that nearly all predictions labeled were accurate. Gradient Boosting (0.84), Random Forest (0.81), and XGBoost (0.84) also performed well, showing consistent and dependable results. Even mid-performing models such as MLP Classifier (0.77) and Logistic Regression (0.74) maintained acceptable precision, while the LDA model, with the lowest score (0.71), suggests a higher rate of false positives.

In Dataset_2, high precision was more broadly distributed across models. AdaBoost (0.88) and Gradient Boosting (0.875) led in performance, followed closely by XGBoost (0.85), Voting Ensemble (0.85), and LGBM (0.85). Most models in this dataset achieved precision above 0.83, reflecting their strong ability to correctly identify peripheral searches with minimal overprediction. These results suggest that, in terms of precision, both datasets support effective classification, with Dataset_2 showing slightly more uniform high performance across models. The precision results for both datasets are presented in detail and summarized in Table 2.

### 4.3. Recall

Recall, also known as Sensitivity or the True Positive Rate, measures a model’s ability to correctly identify all relevant cases—in this context, how many actual peripheral and central eye-tracking instances were correctly detected by the model. It is calculated as the ratio of True Positives to the sum of True Positives and False Negatives. A high recall indicates that the model successfully captures most of the actual peripheral and central location cases, with few missed instances.

The recall results across both datasets reveal how effectively the models identified the true location of the target symbol—whether it appeared in the central or peripheral region of the map—based on eye-tracking metrics. In Dataset_1, several models such as Random Forest, MLP Classifier, SVC, Logistic Regression, and Voting Ensemble achieved high recall values (≥0.81), indicating that these classifiers reliably recognized actual target locations without frequently missing them. Models like Gradient Boosting (0.79) and XGBoost (0.77) also performed well, while others such as AdaBoost (0.72), Decision Tree (0.68), and LDA (0.74) showed a higher rate of false negatives, suggesting they occasionally failed to detect true target areas.

In Dataset_2, recall scores were generally higher, with Random Forest (0.88), LGBM (0.87), and Voting Ensemble (0.86) leading in performance. Even the lower-performing models—such as Logistic Regression (0.70), SVC (0.73), and LDA (0.68)—demonstrated adequate recall, though they were less consistent than ensemble-based methods. This suggests that while Dataset_2 models were more sensitive in detecting actual target locations—whether central or peripheral—they were also more prone to misclassifying cases, likely due to a higher false positive rate. The overall recall results are presented in Table 3.

### 4.4. F1-Score

Classifying whether a participant was searching for a target symbol in the central or peripheral area of the map based on eye-tracking data, the F1-score provides a balanced evaluation of the model’s ability to correctly identify both classes. Specifically, the F1-score is valuable because it combines precision (how many predicted peripheral or central cases were actually correct) and recall (how many actual cases were correctly identified) into a single metric.

For Dataset_1, Gradient Boosting achieved the highest F1-score (0.815), followed closely by Random Forest and Voting Ensemble (both at 0.811), indicating strong and consistent performance across these models. In contrast, the LDA model showed the lowest F1-score (0.722), suggesting that it may struggle to balance precision and recall effectively in this task.

For Dataset_2, Random Forest and LGBM achieved the highest F1-scores (0.858), demonstrating robust classification capabilities. The Gradient Boosting model also performed well, with an F1-score of 0.841. Models like Logistic Regression (0.760) and LDA (0.756) displayed relatively lower F1-scores, indicating that these models might not effectively capture the complexity of the task when distinguishing between peripheral and central areas based on eye-tracking data.

A detailed summary of the results is presented in Table 4.

### 4.5. ROC-AUC

ROC-AUC measures a model’s ability to distinguish between two classes—in this case, central and peripheral search areas. It evaluates how well the model ranks positive instances higher than negative ones across various classification thresholds.

In Dataset_1, the ROC-AUC scores were consistently high across most models, indicating strong classification performance. Voting Ensemble (0.892), XGBoost (0.889), and Random Forest (0.872) achieved the highest scores, suggesting excellent ability to separate the two classes. Other strong performers included LGBM (0.875), Gradient Boosting (0.868), and AdaBoost (0.858). The lowest ROC-AUC was observed for LDA (0.791)—still above random chance but indicating weaker class separation compared to ensemble methods. These results suggest that Dataset_1 is well-suited for identifying peripheral vs. central visual focus, with several models achieving near-excellent separation performance.

In contrast, Dataset_2 produced moderate to lower ROC-AUC scores, reflecting a reduced ability of the models to distinguish between classes. The top scores were achieved by Random Forest (0.736), Voting Ensemble (0.735), and XGBoost (0.729), indicating moderate discriminative power. AdaBoost, Gradient Boosting, and Logistic Regression followed closely (~0.725–0.729), delivering decent, though not exceptional, performance. Lower scores were observed for MLP Classifier (0.647), KNN (0.647), and Decision Tree (0.642), suggesting limited discriminative capacity. Notably, LGBM, despite its strong results on other metrics, exhibited an unexpectedly low ROC-AUC (0.667), possibly due to overfitting or threshold sensitivity in this dataset. Summarized results for both datasets are shown in Table 5.

### 4.6. Feature Importance

Feature importance is provided by models based on decision trees or similar structures that naturally track how features contribute to decision-making [41]. In this research, feature importance is presented for AdaBoost, Gradient Boosting, Random Forest, XGBoost, LightGBM (LGBM), and Decision Tree models. The feature importance analysis across multiple models for Dataset_1 reveals a consistent pattern in which eye-tracking metrics are most influential in classifying visual search behavior as directed toward central or peripheral map areas.

To improve interpretability and generalizability, interaction features were decomposed into their constituent individual features in the feature importance analysis. This approach enables a clearer understanding of each variable’s contribution to model performance.

Fixation count and GazePointY.StandardDeviation consistently rank among the top two most important features across all models, suggesting that both the number of fixations and the vertical gaze dispersion are strong indicators of search behavior. Saccadic count is also highly ranked (third in most models), indicating that the number of saccades adds substantial information for distinguishing between central and peripheral search regions.

The most predictive features in Dataset_1 tend to capture general gaze dynamics (such as fixation and saccade counts) and vertical gaze dispersion (along the *Y*-axis). This suggests that visual searches in central vs. peripheral regions result in measurable differences in gaze clustering and movement, particularly in the vertical dimension. Conversely, features such as pupil variation and fixation skewness, though included, appear to have limited predictive value and may be less useful in future feature selection or model simplification.

The feature importance chart for all models in Dataset_1, including the top five features, is presented in Table 6.

The feature importance results for Dataset_2 reveal consistent patterns in how different gaze-based features contribute to model performance. FixationY.StandardDeviation is the most important feature across all models, often by a wide margin (e.g., over 50% of total importance in Gradient Boosting and the highest absolute gain in LGBM). This strongly suggests that the vertical dispersion of fixation points (GazePointY.StandardDeviation) is a key indicator distinguishing between peripheral and central visual search. GazePointX.StandardDeviation is consistently the second most important feature, indicating that horizontal dispersion also plays a significant role—though typically to a lesser extent than vertical spread.

For Dataset_2, models rely most heavily on the spatial dispersion of fixations—particularly in the Y-direction—to classify search behavior. These findings reinforce the idea that users searching in peripheral areas exhibit more vertically scattered fixations, while central search is likely more concentrated. Additionally, Fixation.Duration Kurtosis, rather than Pupil.StandardDeviation, contributes more to model performance. The feature importance for Dataset_2 is presented in Table 7.

## 5. Discussion

The results of this study align with and build upon previous research in the field of eye tracking and machine learning for cartographic applications. Kiefer et al. [21] demonstrated that eye-tracking data could be used effectively to recognize user activities on maps, achieving a classification accuracy of 78% with a Support Vector Machine (SVM) classifier. This study similarly explores how eye-tracking features can be used to predict user behavior during map reading tasks, specifically targeting the location of a symbol in the central or peripheral areas. While Kiefer et al.’s work focused on differentiating between activities like exploration and route planning, this study offers a more granular analysis of the user’s visual search process, using eye-tracking metrics to predict the location of a symbol on a map, which might be applicable for the automatic matching of eye-tracking data with map features such as object location [42].

Liao et al. [22] further demonstrated the applicability of machine learning in predicting user tasks, with a random forest classifier achieving an accuracy of 67% in pedestrian navigation scenarios. While their study focused on task classification based on eye movement features such as fixation density and saccade encoding, this study takes a more focused approach by predicting the location of a target symbol during a visual search. Both studies underscore the importance of saccade and fixation-related features in distinguishing between different user behaviors. Fixation count values have been previously evaluated as an important factor in understanding a user’s map reading strategies [12,43].

In this study, gaze dispersion, especially GazePointY.StandardDeviation, proved to be a more influential factor in predicting target symbol locations compared to saccadic features. This observation contrasts with findings from Qin et al. [23], who highlighted saccadic metrics as key in classifying map activities. While saccadic features did play an important role in distinguishing between peripheral and central locations in this research, the impact of vertical gaze dispersion was more pronounced. This difference suggests that standard deviation across the Y axis may provide a more reliable indicator of user attention and spatial focus during visual search tasks. This is also related to the findings of Dong et al. [19], which suggest that higher fixation dispersion is experienced by non-geographers as participants, which might indicate spatial abilities as the crucial factor or task difficulty [20].

While the actual location of a map symbol is visible, predicting it from eye movements offers insight into user cognition, attention, and interaction patterns. These findings show that vertical gaze dispersion and fixation count reflect not only spatial search behavior but also user engagement and adaptive strategies—especially in systems unaware of the target’s position, such as exploratory or assistive map interfaces. Classifying central versus peripheral search from gaze data reveals implicit user states, enabling timely adaptations (e.g., symbol enhancement, map scaling). Repeated peripheral search difficulties may inform user modeling, allowing for personalized support or design adjustments.

## 6. Conclusions

In comparing the results across both datasets, Dataset 1 generally demonstrates superior model performance, particularly in terms of accuracy and class separation. Models such as AdaBoost and Gradient Boosting performed exceptionally well, achieving high accuracy (0.822) and strong precision (AdaBoost at 0.90), indicating that these models are highly reliable in making correct predictions. Moreover, the ROC-AUC scores for Dataset 1 were consistently high, with the Voting Ensemble (0.892) and XGBoost (0.889) showing excellent discrimination between the central and peripheral search areas. This suggests that Dataset 1 has more distinct patterns and informative features, making classification easier and more accurate. The models in Dataset 1 are also better at achieving a balance between precision and recall, as seen in the high F1-scores, particularly from Gradient Boosting (0.815) and Random Forest (0.811). These findings highlight the robustness of the dataset in terms of the models’ ability to make reliable predictions with few errors.

Conversely, Dataset 2 demonstrated more variability in model performance, with lower overall accuracy and ROC-AUC scores. While LGBM performed reasonably well with an accuracy of 0.773 and precision scores above 0.80, the model struggled with class separation, as evidenced by the relatively low ROC-AUC score (0.667). The models in Dataset 2 also exhibited more inconsistencies in their recall, with Random Forest (0.88) and LGBM (0.87) performing well, but other models like Logistic Regression and LDA showing lower recall and precision. This suggests that Dataset 2 is more challenging for classification tasks, likely due to either less informative features or more complex patterns that the models struggle to capture. Overall, Dataset 2 requires more attention to feature engineering and model optimization to improve class separation and recall.

An important finding concerns the role of specific eye-tracking features, particularly fixation count and GazePointY.StandardDeviation, which emerged as highly important predictors. A higher fixation count may reflect increasing familiarity with the visual stimulus, as users accumulate information through repeated fixations. This accumulation likely facilitates locating the target symbol, making it easier to predict whether it lies in the central or peripheral area of the map. Similarly, higher vertical gaze dispersion may indicate broader vertical scanning behavior, which could be associated with search strategies when exploring peripheral areas or integrating map elements across vertical extents. These interpretations align well with known cognitive processes in visual search and spatial exploration and highlight meaningful avenues for deeper investigation.

However, several limitations of this study should be acknowledged. First, the sample size and participant diversity may limit the generalizability of the findings to broader user populations with varying levels of map-reading expertise or cognitive abilities. Second, the experimental setting involved controlled visual search tasks, which may not fully capture the complexity and variability of real-world map interaction scenarios. Third, while the selected eye-tracking metrics, including fixation duration, saccadic count, and gaze dispersion, provided valuable insights, other potentially informative features—such as pupil dilation, fixation sequence patterns, or contextual task variables—were not incorporated into the models. Additionally, while machine learning models showed promising performance, particularly for Dataset 1, the lower performance for Dataset 2 indicates that further feature engineering, model tuning, and potentially the inclusion of more advanced deep learning approaches could improve classification robustness, especially under more complex conditions.

Future research should address these limitations by expanding participant samples, incorporating more ecologically valid tasks that reflect real-world map usage, and exploring additional eye-tracking features and multimodal data sources. Investigating temporal dynamics of gaze behavior and integrating contextual information from user tasks could also enhance predictive accuracy. Moreover, longitudinal studies could examine how user expertise and learning influence gaze dispersion patterns over time. Ultimately, advancing these approaches may contribute to the development of intelligent, gaze-adaptive cartographic systems that dynamically respond to individual user needs and optimize map usability in diverse application domains.

In conclusion, the findings from this study highlight the potential of machine learning models to predict the location of target symbols in map reading tasks based on eye-tracking data. By analyzing eye movement parameters such as fixation duration, saccade length, and gaze point dispersion, this study demonstrates how these metrics can be leveraged to classify whether a symbol appears in the central or peripheral area of the map. This ability to accurately predict symbol locations has a potential relevance for improving the design of dynamic, user-adaptive maps. By tailoring map displays in real time based on the user’s gaze behavior, future map applications can enhance the user experience by improving search efficiency, engagement, and overall usability. The results underscore the value of integrating cognitive models with eye-tracking technology, offering new avenues for optimizing cartographic design to better suit the visual search strategies and cognitive needs of map users.

## Figures and Tables

**Table 1:** Model accuracy results for both datasets used in the study. The juxtaposition clearly shows that Dataset_1 appears better suited for predicting peripheral and central symbol locations compared to Dataset_2.

Model	Dataset_01 Accuracy	Dataset_02 Accuracy
AdaBoost	0.82	0.72
Gradient Boosting	0.82	0.76
Random Forest	0.81	0.77
XGBoost	0.81	0.76
Voting Ensemble	0.81	0.77
LGBM	0.80	0.77
MLP Classifier	0.78	0.74
Logistic Regression	0.77	0.65
KNN	0.76	0.72
SVC	0.76	0.68
Decision Tree	0.75	0.71
LDA	0.72	0.65

**Table 2:** Model precision results for both datasets used in the study. The juxtaposition suggests that, in terms of precision, both datasets support effective classification, with Dataset_2 showing slightly more uniform high performance across models.

Model	Dataset_01 Precision	Dataset_02 Precision
AdaBoost	0.90	0.88
Gradient Boosting	0.84	0.88
Random Forest	0.81	0.84
XGBoost	0.84	0.85
Voting Ensemble	0.81	0.85
LGBM	0.82	0.85
MLP Classifier	0.77	0.82
Logistic Regression	0.74	0.83
KNN	0.75	0.84
SVC	0.73	0.84
Decision Tree	0.78	0.84
LDA	0.71	0.84

**Table 3:** Model recall results for both datasets used in the study. These findings suggest that Dataset_1 offers more discriminative and consistent features, making it better suited for robust classification, whereas Dataset_2 supports more sensitive detection, potentially at the cost of generalization accuracy.

Model	Dataset_01 Recall	Dataset_02 Recall
AdaBoost	0.72	0.74
Gradient Boosting	0.79	0.81
Random Forest	0.81	0.88
XGBoost	0.77	0.85
Voting Ensemble	0.81	0.86
LGBM	0.77	0.87
MLP Classifier	0.81	0.86
Logistic Regression	0.81	0.70
KNN	0.75	0.79
SVC	0.81	0.73
Decision Tree	0.68	0.78
LDA	0.74	0.68

**Table 4:** Model F1-score results for both datasets used in the study. Overall, the F1-scores suggest that while many models perform well across both datasets, Random Forest, LGBM, and Gradient Boosting consistently stand out, offering a strong balance between precision and recall.

Model	Dataset_01 F1-Score	Dataset_02 F1-Score
AdaBoost	0.80	0.80
Gradient Boosting	0.815	0.84
Random Forest	0.81	0.86
XGBoost	0.80	0.85
Voting Ensemble	0.81	0.86
LGBM	0.80	0.86
MLP Classifier	0.79	0.84
Logistic Regression	0.77	0.76
KNN	0.75	0.81
SVC	0.77	0.78
Decision Tree	0.73	0.81
LDA	0.722	0.76

**Table 5:** Model ROC-AUC results for both datasets used in the study. Dataset_1 demonstrates stronger class separation across a wide range of models, making it more effective for visual search classification tasks.

Model	Dataset_01 ROC-AUC	Dataset_02 ROC-AUC
AdaBoost	0.86	0.725
Gradient Boosting	0.87	0.73
Random Forest	0.87	0.735
XGBoost	0.89	0.73
Voting Ensemble	0.89	0.73
LGBM	0.87	0.67
MLP Classifier	0.84	0.65
Logistic Regression	0.82	0.73
KNN	0.82	0.65
SVC	0.87	0.66
Decision Tree	0.81	0.64
LDA	0.79	0.65

**Table 6:** Feature importance across decision tree models for Dataset_1. Composite (interaction) features were decomposed to highlight the underlying individual features that consistently contribute to model performance across various models.

Feature	AdaBoost	Gradient Boosting	Random Forest	XGBoost	LGBM	Decision Tree
Fixation.Count	0.31	0.30	0.28	0.30	0.29	0.28
GazePointY.StandardDeviation	0.25	0.35	0.29	0.25	0.24	0.29
Saccadic.Count	0.19	0.17	0.21	0.25	0.20	0.21
GazePointX.StandardDeviation	0.10	0.09	0.09	0.09	0.12	0.10
Fixation.Duration.Skewness	0.08	0.03	0.07	0.06	0.08	0.07
Pupil.StandardDeviation	0.07	0.05	0.04	0.04	0.07	0.05

**Table 7:** Feature importance across decision tree models for Dataset_2. In this dataset, Fixation.Duration Kurtosis is the fourth most important feature.

Feature	AdaBoost	Gradient Boosting	Random Forest	XGBoost	LGBM	Decision Tree
GazePointY.StandardDeviation	0.46	0.54	0.42	0.41	0.36	0.43
GazePointX.StandardDeviation	0.24	0.26	0.24	0.26	0.26	0.27
Fixation.Duration.Skewness	0.10	0.06	0.09	0.10	0.11	0.09
Fixation.Duration.Kurtosis	0.08	0.06	0.08	0.08	0.10	0.08
Saccadic.Count	0.07	0.05	0.08	0.07	0.09	0.07
Fixation.Count	0.05	0.03	0.08	0.07	0.07	0.05

## Data Availability

Data that supports this study is based on two studies: Cybulski, P.; Krassanakis, V. The effect of map label language on the visual search of cartographic point symbols. *Cartogr. Geogr. Inf. Sci.* **2022**, 49(3), 189–204. https://doi.org/10.1080/15230406.2021.2007419 [25]—available on request; and Cybulski, P.; Ledermann, F. The impact of point symbol similarity on visual search on maps. *Cartogr. Geogr. Inf. Sci.* **2025**, 52(4), 423–440. https://doi.org/10.1080/15230406.2024.2409166 [26]—openly available https://doi.org/10.7910/DVN/IUK4SZ.
